# Identification of Novel Rare *ABCC1* Transporter Mutations in Tumor Biopsies of Cancer Patients

**DOI:** 10.3390/cells9020299

**Published:** 2020-01-26

**Authors:** Onat Kadioglu, Mohamed Saeed, Markus Munder, Andreas Spuller, Henry Johannes Greten, Thomas Efferth

**Affiliations:** 1 Department of Pharmaceutical Biology, Institute of Pharmacy and Biochemistry, Johannes Gutenberg University, 55128 Mainz, Germany; kadioglu@uni-mainz.de (O.K.); saeedm@uni-mainz.de (M.S.); 2 Third Department of Medicine (Hematology, Oncology, and Pneumology), University Medical Center of the Johannes Gutenberg University Mainz, 55131 Mainz, Germany; markus.munder@unimedizin-mainz.de; 3 Clinic for Gynecology and Obstetrics, 76131 Karlsruhe, Germany; andreas-spuller@web.de; 4 Abel Salazar Biomedical Sciences Institute, University of Porto, 4099-030 Porto, Portugal; heidelbergschool@aol.com; 5 Heidelberg School of Chinese Medicine, 69126 Heidelberg, Germany

**Keywords:** ABC transporters, cancer, multidrug resistance

## Abstract

The efficiency of chemotherapy drugs can be affected by ATP-binding cassette (ABC) transporter expression or by their mutation status. Multidrug resistance is linked with ABC transporter overexpression. In the present study, we performed rare mutation analyses for 12 *ABC* transporters related to drug resistance (ABCA2, -A3, -B1, -B2, -B5, -C1, -C2, -C3, -C4, -C5, -C6, -G2) in a dataset of 18 cancer patients. We focused on rare mutations resembling tumor heterogeneity of ABC transporters in small tumor subpopulations. Novel rare mutations were found in *ABCC1*, but not in the other ABC transporters investigated. Diverse *ABCC1* mutations were found, including nonsense mutations causing premature stop codons, and compared with the wild-type protein in terms of their protein structure. Nonsense mutations lead to truncated protein structures. Molecular docking and heat map analyses of *ABCC1*/MRP1 pointed out that Lys498* appeared in a separate cluster branch due to the large deletion, leading to a massive disruption in the protein conformation. The resulting proteins, which are nonfunctional due to nonsense mutations in tumors, offer a promising chemotherapy strategy since tumors with nonsense mutations may be more sensitive to anticancer drugs than wild-type *ABCC1*-expressing tumors. This could provide a novel tumor-specific toxicity strategy and a way to overcome drug resistance.

## 1. Introduction

ATP-binding cassette (ABC) transporters are drug efflux pumps hampering the effectiveness of cytotoxic anticancer drugs. ABC transporter overexpression is associated with multidrug resistance (MDR) in cancer cells, possibly leading to chemotherapy failure [1]. The overexpression of ABC transporters is also associated with shorter survival rates of cancer patients [2,3,4]. RNA-sequencing and other genome- and transcriptome-wide techniques paved the way to address the MDR phenomenon and the corresponding influence of ABC transporters in a more comprehensive fashion.

The advances in next-generation sequencing methodology have spurred researchers to study a large amount of genomic data to identify patients’ tumor-specific mutations. Indeed, this knowledge will assist clinicians in making personalized treatment decisions to improve treatment strategies [5,6]. In recent years, medical oncologists have focused on targeted sequencing to identify genetic aberrations that may be predictive of response to anticancer therapies [7]. While most analyses focused on mutation profiling of the main cell populations of a tumor, the mutations in small subpopulations may be especially decisive for the refractoriness of tumors. Tumor cells with mutations in drug resistance genes may survive and grow if the predominant tumor population has been eradicated by chemotherapy. Tumor heterogeneity within a patient has been recognized as a major factor of drug resistance [8,9]. It is worth mentioning that the advancement of genome-wide DNA mutation analyses have enabled the identification of hundreds of low-frequency mutated genes in tumors and triggered scientists to unravel their impact on the prognosis and pathogenesis of cancer [10]. Structural comparisons of truncated forms of *ABC* transporters with wild-type proteins have shed light on the effect of these nonsense mutations. Nonsense mutations were clearly associated with truncated protein structures. Tumors carrying nonsense mutations in ABC genes possess nonfunctional proteins for the corresponding ABC transporter. The determination of truncated nonfunctional ABC transporters may imply a promising chemotherapy strategy. Tumors with nonsense mutations in ABC transporters may be sensitive to chemotherapy with ABC transporter substrates. While tumors carry a nonsense-mutated ABC transporter, this transporter is not mutated in normal tissues and is still intact. Hence, chemotherapy would preferentially affect tumor tissues with nonsense-mutated and nonfunctional ABC transporters rather than normal tissues. This strategy may trigger a novel tumor-specific chemotherapy strategy to overcome drug resistance. We analyzed low-frequency mutations in 12 ABC transporters associated with drug resistance (ABCA2, -A3, -B1, -B2, -B5, -C1, -C2, -C3, -C4, -C5, -C6, -G2) [11,12,13,14,15]. Novel *ABCC1* transporter mutations, including nonsense mutations causing premature stop codons, were identified that have not been reported before.

In the present study, we performed RNA-sequencing in tumors from 16 patients with different tumor types at a late stage who had not responded to conventional chemotherapy and two leukemia patients’ biopsies were collected during the initial diagnosis (n = 18 in total). We specifically focused on low-frequency mutations. Additionally, we identified novel nonsense and missense mutations in the *ABCC1* gene and speculate that substrates of MDR-associated protein 1 (MRP1, encoded by the *ABCC1* gene), such as doxorubicin, docetaxel, etoposide, and teniposide could be administered to patients with nonsense *ABCC1* mutations. Furthermore, we selected three missense and one nonsense mutations, in order to evaluate the binding mode of MRP1 substrates and inhibitors. By applying heat map analyses, we compared the binding patterns with those of wild-type MRP1.

## 2. Material and Methods

### 2.1. RNA Sequencing and Mutation Analysis

The ABC transporter mutations in our dataset of 18 patients with various cancer types were identified by RNA sequencing. Informed consent was collected from all patients. The procedure of RNA sequencing has been described previously [16]. The clinical data of the patients is described in Table 1. Considering frequent mutations, none of the patients possess nonsense mutations. In order to identify the low frequent mutations, Strand NGS 3.4 software (Strand Life Sciences Pvt. Ltd., Bangalore, India) was used. Twelve ABC transporters together with their chromosomal position were selected and imported as a gene list. As a first step, the patients’ “.vcf” files and a “.bam” file as a reference human genome alignment were imported. Then, by using the “filter by region list” option, read lists (aligned reads) and region lists (patient data) were selected to generate a further read list. The next round of “filter by region list” was performed by selecting the read list from the previous step and the imported ABC transporter gene list as the region list. This final read list was used to perform low-frequency SNP detection by clicking on “SNP detection” and “perform low frequency SNP detection” with default options. Default lower threshold of the base quality range for the binomial test iteration is 20 and default upper threshold of the base quality range for the binomial test iteration is 30 for low-frequency SNP detection. Detailed explanation for low-frequency SNP detection is listed at the user manual Section 11.5.4 of Strand NGS software. We took the same threshold for low-frequency mutations. Afterwards, SNP effect analysis was performed, and the gene lists and the mutations were exported.

### 2.2. Homology Modeling, Protein Structure Preparation

A human MRP1 homology model was created by using bovine Mrp1 (PDB ID: 5UJ9) as template. The Modeller 9.23 algorithm embedded in UCSF Chimera software was used as previously described [17]. Structural comparisons were performed for human MRP1 focusing on nonsense mutations which cause premature stop codons. Corresponding protein structure files were prepared by using the human MRP1 homology model.

### 2.3. Molecular Docking and Clustering

The modeled wild-type MRP1, three missense mutations (T550A, T556A, and V1101F) and one nonsense mutation (Lys498*) were subjected to molecular docking analyses against a panel of MRP1 substrates (zoledronic acid, irinotecan, etoposide, epirubicin, doxorubicin, docetaxel, dactinomycin, 7-ethyl-10-hydroxycamptothecin, and camptothecin) and MRP1 inhibitors (reversan, mk-571, glibenclamide, pak104p, sulfinpyrazone, indomethacin, probenecid, quercetin, genistein, and diltiazem) [18,19,20,21,22,23,24,25,26,27,28]. The two missense mutations, T550A and T556A, were hotspot mutations in the transmembrane domain. Thr550 and Thr556 were located in the inner leaflet region of transmembrane domain 10 (TM10), playing a vital role in determining the drug resistance profile [29]. The V1101F mutation was also observed in COSMIC database, present in the HCT15 colon cancer cell line. Lys498* was the nonsense mutation observed in our patients leading to the largest deletion in MRP1. A total of four human MRP1 homology models, 9 classical anticancer drugs that are MRP1 substrates, and 10 MRP1 inhibitors have been subjected to an automated and comprehensive virtual molecular docking campaign. Each molecular docking was based on at least three independent dockings, each consisting of 2,500,000 calculations. The AutoDock 4 algorithm was used for the defined molecular docking calculations on the drug-binding pocket of MRP1 as previously described [30].

Hierarchical clustering was performed with the CIM miner software (https://discover.nci.nih.gov/cimminer/home.do) (Bethesda, MD, USA) as previously described by applying ward cluster algorithm [31,32]. The heat maps were visualized in order to classify the compounds and the mutant models in terms of their binding energy distributions.

## 3. Results

### 3.1. ABC Transporters Mutation Analysis

Low-frequency missense and nonsense mutations were only observed for *ABCC1*, but not for the other 11 ABC transporters investigated. These low-frequency nonsense mutations in *ABCC1* are listed in Table 2. Low-frequency missense and deletion/insertion mutations in *ABCC1* are listed in Table 3. All identified nonsense mutations in our patient dataset are new and were not listed in the COSMIC database (https://cancer.sanger.ac.uk/cosmic/gene/analysis?ln=ABCC1#variants). Therefore, they can be considered as novel mutations.

**Figure 1:**
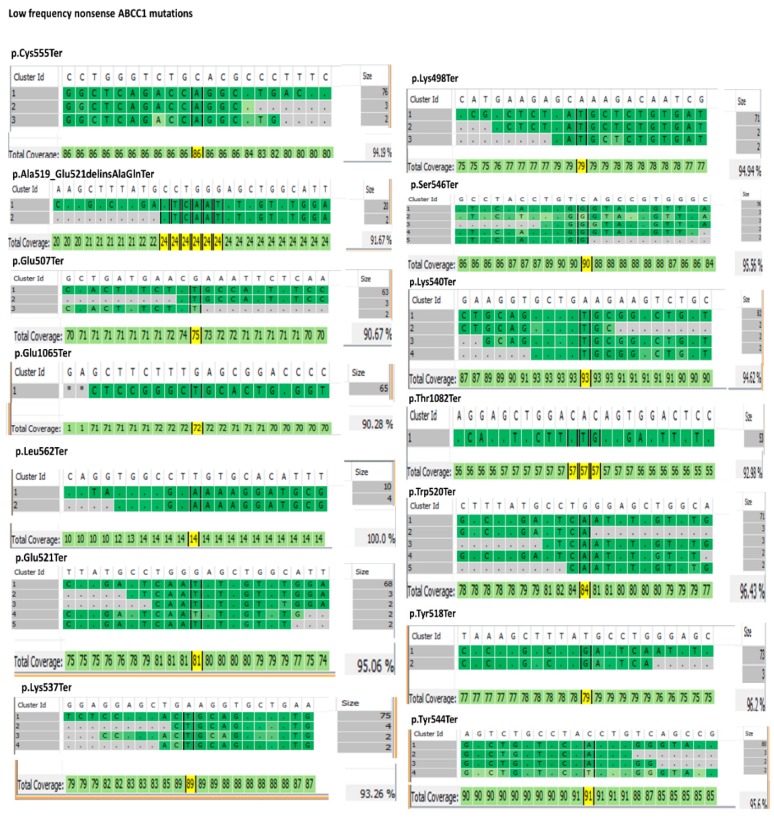
Low-frequency nonsense *ABCC1* mutation sequence reads and the corresponding alignments. Reference genome sequence (gray) is on top of each screenshot. Sequence reads from the tumor biopsy with different clusters are labeled in green. Read numbers of each cluster sequence for that region are shown on the right side of each screenshot. On the bottom right side, the coverage percentage for the shown cluster reads is depicted for that region. One patient for each mutation was selected for visualization.

For visualization of these low-frequency nonsense mutations, their read values and the corresponding alignments are depicted in Figure 1.

### 3.2. Structural Comparison of the Nonsense MRP1 Mutant Models

**Figure 2:**
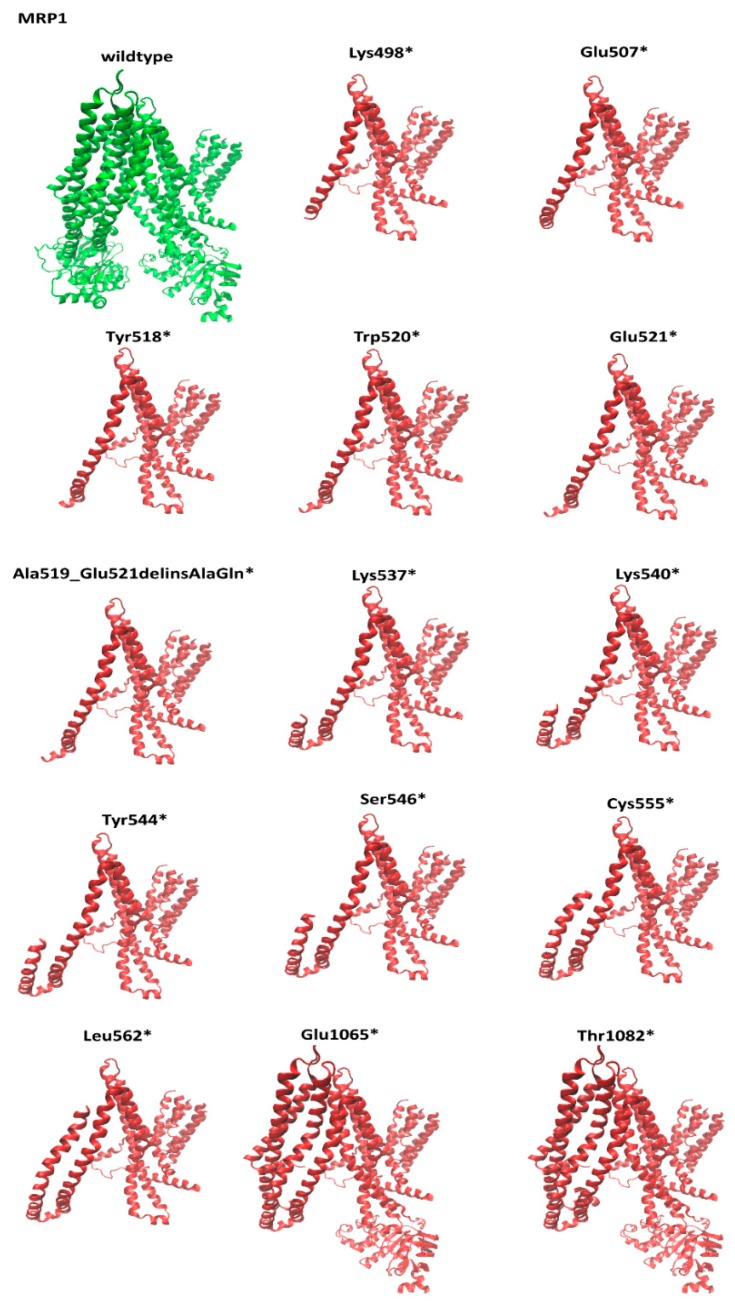
Influence of nonsense mutations on the *ABCC1* gene and structural comparison with wild-type MRP1 protein structure. Wild-type MRP1 protein (green) and mutant MRP1 homology models (red).

Low-frequency *ABCC1* nonsense mutation caused truncated MRP1 protein structures. A structural comparison of each low-frequency nonsense mutant with the wild-type MRP1 structure is visualized in Figure 2.

### 3.3. Molecular Docking Clustering

**Figure 3:**
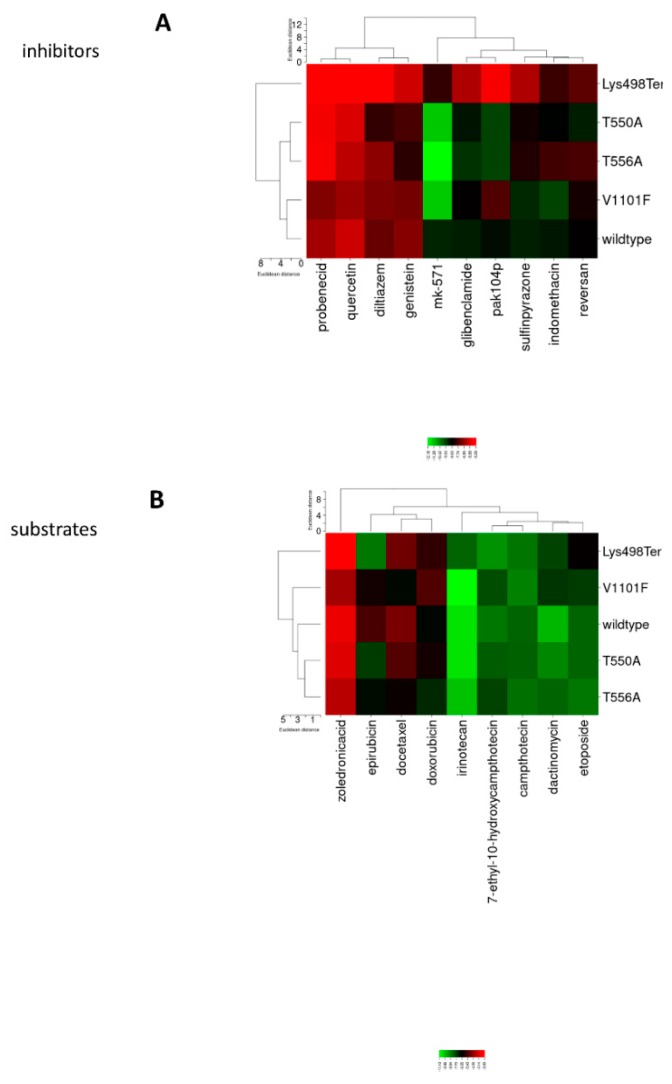
Heat maps for the docking analyses of (**A**) MRP1 inhibitors, (**B**) MRP1 substrates (green color: strong interaction; red color: weak interaction) on wild-type and mutant MRP1.

Hierarchical clustering and heat maps are visualized for MRP1 inhibitors in Figure 3A and substrates in Figure 3B. For both inhibitors and substrates, Lys498* appeared in a separate cluster branch as expected due to the large deletion, leading to a massive disruption in the conformation. T550A and T556A mutants clustered together on the heat map for both inhibitors and substrates, indicating that both mutants have the same pattern of conformation. Interestingly, the V1101F mutant clustered with the wild type for inhibitors and clustered in close distance for substrates, suggesting this mutant has little effect on the MRP1 transport function. The inhibitor Mk-571 showed higher affinity to the mutants than the wild type. Glibenclamide and pak104p revealed lower binding energies for the T550A and T556A mutants than for the wild type and V1101F mutant. Intriguingly, epirubicin showed higher affinity to the nonsense-mutated and truncated MRP1 than the wild type and the other mutants. This could be attributed to a unique binding site that might be reachable for epirubicin in the deleted form of MRP1.

## 4. Discussion

The development of MDR phenotypes is a major obstacle in the treatment of malignancies as it leads to therapeutic failure. Overexpression of *ABCC1* is considered as one of the mechanisms that elicits MDR development in cancer cells due to its capacity to expel the chemotherapeutic drugs from the cells [33]. MRP1 localizes primarily at the plasma membrane of the cells and in the membranes of sub-cellular organelles such as mitochondria, endoplasmic reticulum and endocytic vesicles [34]. *ABCC1* mRNA overexpression has been reported in small-cell lung carcinoma [35], prostate [36], neuroblastoma [37], acute lymphoblastic leukemia [38], breast [39], and glioblastoma [25].

In the present study, we performed rare mutation analyses for 12 *ABC* transporters related to drug resistance (ABCA2, -A3, -B1, -B2, -B5, -C1, -C2, -C3, -C4, -C5, -C6, -G2) in a dataset consisting of RNA sequencing data from 18 cancer patients. Novel rare mutations were found only in *ABCC1*. Low-frequency *ABCC1* mutations have been identified, which reflect tumor heterogeneity and the presence of various missense and nonsense mutations at the *ABCC1* coding region for each patient. Cases with a large deletion as result of a nonsense mutation were characterized by dramatic structural changes. These kind of nonsense mutations presumably led to aberrant structures and thus nonfunctional or only partially functional MRP1 transporters. As a result, MRP1 substrates could not be recognized by the transporter anymore and could remain within the tumor cells without being expelled. This implies a novel personalized chemotherapy approach in those cases, where nonsense mutations in *ABCC1* are determined. Tumors with nonfunctional/partially functional MRP1 due to the presence of premature stop codon formations may be more sensitive towards anticancer substrates of MRP1, but normal tissues expressing wild-type *ABCC1*/MRP1 are not sensitized at the same time, because these nonsense mutations occur as somatic mutations only in tumor cells, but not as germline mutations in normal tissues. The identification of *ABCC1*/MRP1 nonsense mutations, therefore, could be critical in this regard and could take place on an individual basis by sequencing each tumor transcriptome.

All identified nonsense mutations in our patient dataset are novel. These results considerably expand upon the findings of Yin et al. [40] in analyzing the role of MRP1 polymorphisms in drug resistance, toxicity, and prognosis prediction. They identified only 14 non-synonymous polymorphisms with very low frequencies, but no nonsense polymorphism has been found [40]. Single nucleotide polymorphisms occur in healthy tissues at variable high percentages and may therefore also occur in tumors derived from these normal tissues. The results we obtained in our analysis are somatic mutations in tumors. Saito et al. reported 95 genetic variations in *ABCC1* out of the identified 779 genetic variations in eight ABC genes in the Japanese population; only one of them is a missense mutation (R723Q) and none of them are nonsense mutations [41]. Fukushima-Ueseka et al. identified 86 genetic variations in *ABCC1*, including 31 novel variations in the same population, of which 11 were missense and none were nonsense mutations [42]. Leschziner et al. screened five ABC transporter genes in a Caucasian population and observed 61 out of 221 variations are in *ABCC1*, among which 22 are novel [43]. In our study, we focused on rare missense, del/ins, and nonsense mutations. We identified 14 novel, rare *ABCC1* nonsense mutations (Table 2) and 301 novel, rare *ABCC1* missense and del/ins mutations (Table 3).

As shown by our molecular docking results, the truncated *ABCC1*/MRP1 forms revealed different patterns of drug binding than the wild type and other mutants for specific MRP1 inhibitors and substrates, because they clearly appeared as a separate cluster. Importantly, the known MRP1 inhibitor, Mk-571, showed higher affinity to missense mutations than wild-type MRP1. Combining MRP1 chemotherapeutic substrates with Mk-571 might be a favorable therapeutic option for patients whose *ABCC1* gene bears these missense mutations.

The two polar residues, Thr550 and Thr556, located in the inner leaflet region of TM10, play a vital role in determining the drug resistance profile [29]. Both residues were mutated and led to an amino acid exchange and placement of alanine. Increased resistance to vincristine and decreased resistance to doxorubicin and etoposide were observed in the T550A and T556A mutations which was attributed to the smaller size of the Ala side chain that favors transport of the larger drug [29]. Moreover, our heat map analyses based on the molecular docking results clearly showed that these two missense mutations were clustered together and had the same pattern of substrate/inhibitor bindings. To the best of our knowledge, this is the first time to report those two missense mutations in clinical samples. Since both mutations are determinants for drug resistance, this warrants further care in the selection of chemotherapeutic agents for patients bearing these mutations.

In conclusion, a common assumption is that the low-frequency-mutation analyses may have more prognostic significant values for driver mutations [44]. Hence, mutations in driver genes have been mainly in the spotlight. In the present investigation, we intentionally did not focus on driver mutations that are important for carcinogenesis and tumor progression. Instead, we concentrated on ABC transporters as important mediators of chemotherapeutic drug resistance. In fact, we found that pointing to nonsense mutations in ABC transporters may offer a unique opportunity for precision medicine. Nonsense mutations in ABC transporters lead to truncated and nonfunctional proteins offering unique chances to sensitize tumor patients to chemotherapy. RNA-sequencing of tumor transcriptomes on an individual patient-to-patient basis may, therefore, unravel opportunities to successfully treat otherwise refractory cancer patients with drugs that are substrates of specific ABC transporters.

Furthermore, rare mutations contribute to intratumoral heterogeneity and sub-clonal diversity for primary and metastatic tumors. Therefore, identification of rare mutations in ABC transporters, coupled with knowledge of protein structure and functional insight, may allow the design of novel and appropriate strategies for individualized cancer treatment.

## Figures and Tables

**Table 1:** Patient Information.

Patient ID	Tumor Type	Age	Gender
P01	vulva cancer, colon cancer	82	Female
P02	breast	64	Female
P03	breast	75	Female
P04	lung	75	Female
P05	breast	76	Female
P06	non-Hodgkin lymphoma	56	Male
P07	non-Hodgkin lymphoma	68	Male
P08	lymphoma	43	Female
P09	breast	50	Female
P10	cholangiocellular cancer of bile duct	54	Male
P11	liver	80	Male
P12	cervix	54	Female
P13	invasive lobular breast cancer	50	Female
P14	pancreas cancer	62	Male
P15	squamous epithelium cancer of the base of the tongue	57	Male
P16	acute myeloid leukemia	64	Female
P17	serous adeno cancer of the tube	57	Female
P18	acute myeloid leukemia	82	Male

**Table 2:** Low frequent nonsense mutations in *ABCC1*.

Mutation	Patient
Ala519_Glu521delinsAlaGlnTer	P16, P03, P04, P17, P08
Cys555Ter	P16, P03, P13, P08, P15
Glu1065Ter	P16, P01, P02, P03, P05, P06, P13, P08, P11, P09, P10
Glu507Ter	P03, P04, P17, P08
Glu521Ter	P16, P03, P04, P17, P13, P08
Leu562Ter	P16, P18, P02, P03, P05, P06, P17, P13, P08, P11, P12, P15, P14, P09
Lys498Ter	P03, P04, P17, P08
Lys537Ter	P16, P03, P17, P13, P08
Lys540Ter	P16, P03, P17, P13, P08
Ser546Ter	P16, P03, P13, P08
Thr1082Ter	P16, P01, P02, P03, P05, P06, P07, P13, P08, P11, P09, P10
Trp520Ter	P16, P03, P04, P17, P08
Tyr518Ter	P16, P03, P04, P17, P08
Tyr544Ter	P16, P03, P13, P08

**Table 3:** Low frequent missense and del/ins mutations in *ABCC1*.

Mutation	Patient	Mutation	Patient	Mutation	Patient
Ala1104Asp	P16, P01, P03, P04, P07, P17, P13, P08, P11, P12	His494Leu	P03, P04, P17, P08	Phe1064Cys	P16, P01, P02, P03, P05, P06, P13, P08, P11, P09, P10
Ala493Ser	P03, P04, P17, P08	His494Phe	P03, P04, P17, P08	Phe1064Val	P16, P01, P02, P03, P05, P06, P13, P08, P11, P09, P10
Ala519Thr	P16, P03, P04, P17, P08	His494Tyr	P03, P04, P17, P08	Phe1076Leu	P16, P01, P02, P03, P05, P06, P07, P13, P08, P11, P09, P10
Ala523_Asp526delinsValGluLeuSer	P16, P03, P04, P17, P13, P08	His701Pro	P01, P01, P03, P03, P07, P07, P13, P13, P08, P08, P15, P15	Phe1076Ser	P16, P01, P02, P03, P05, P06, P07, P13, P08, P11, P09, P10
Ala523Val	P16, P03, P04, P17, P13, P08	Ile1087Leu	P16, P01, P02, P03, P05, P06, P07, P13, P08, P11, P09, P10	Phe1094_Gly1096delinsTrpSerGln	P16, P01, P02, P03, P05, P06, P07, P13, P08, P11, P09, P10
Ala530_Ile531delinsGlyHis	P16, P03, P17, P13, P08	Ile1087Thr	P16, P01, P02, P03, P05, P06, P07, P13, P08, P11, P09, P10	Phe1094Cys	P16, P01, P02, P03, P05, P06, P07, P13, P08, P11, P09, P10
Ala530Gly	P16, P03, P17, P13, P08	Ile1091_Lys1092delinsMetGlu	P12	Phe1094Leu	P16, P01, P02, P03, P05, P06, P07, P13, P08, P11, P12, P09, P10
Ala543Val	P16, P03, P13, P08	Ile1091Asn	P16, P01, P02, P03, P05, P06, P07, P13, P08, P11, P09, P10	Phe1099Leu	P16, P01, P03, P07, P13, P08, P11, P12
Ala547Asp	P16, P03, P13, P08	Ile1091Met	P16, P01, P02, P03, P05, P06, P07, P13, P08, P11, P12, P09, P10	Phe1099Ser	P16, P01, P03, P07, P13, P08, P11, P12
Ala547Ser	P16, P03, P13, P08	Ile1091Val	P16, P01, P02, P03, P05, P06, P07, P13, P08, P11, P09, P10	Phe314Ala	P07, P13, P10
Ala693_Glu694delinsMetLeu	P01, P03, P07, P13, P08, P15	Ile1102Leu	P16, P01, P03, P04, P07, P17, P13, P08, P11, P12	Phe314Ser	P07, P13, P10
Ala693Thr	P01, P03, P07, P13, P08, P15	Ile1102Pro	P16, P01, P03, P04, P07, P17, P13, P08, P11, P12	Phe314Val	P07, P13, P10
Ala693Val	P01, P03, P07, P13, P08, P15	Ile1102Thr	P16, P01, P03, P04, P07, P17, P13, P08, P11, P12	Phe321Val	P07, P13, P10
Ala703Pro	P01, P03, P07, P13, P08, P15	Ile345Leu	P04, P07, P13, P10	Phe325Leu	P07, P13, P10
Ala703Val	P01, P03, P07, P13, P08, P15	Ile508Asn	P03, P04, P17, P08	Phe524Leu	P16, P03, P04, P17, P13, P08
Arg1066Pro	P16, P01, P02, P03, P05, P06, P13, P08, P11, P09, P10	Ile508Leu	P03, P04, P17, P08	Phe524Tyr	P16, P03, P04, P17, P13, P08
Arg1075_Ser1077delinsLysProGly	P16, P01, P02, P03, P05, P06, P07, P13, P08, P11, P09, P10	Ile512_Lys513delinsArgGlu	P16, P03, P04, P17, P08	Phe524Val	P16, P03, P04, P17, P13, P08
Arg1075His	P16, P01, P02, P03, P05, P06, P07, P13, P08, P11, P09, P10	Ile512_Lys513delinsSerGlu	P03, P08	Phe551Ser	P16, P03, P13, P08, P15
Arg1075Ser	P16, P01, P02, P03, P05, P06, P07, P13, P08, P11, P09, P10	Ile512Ser	P16, P03, P04, P17, P08	Phe558Ile	P16, P03, P13, P08, P15
Arg501Leu	P03, P04, P17, P08	Ile531Asn	P16, P03, P17, P13, P08	Phe558Ser	P16, P03, P13, P08, P15
Arg532Met	P16, P03, P17, P13, P08	Ile531Leu	P16, P03, P04, P17, P13, P08	Phe565Leu	P16, P18, P02, P03, P05, P06, P17, P13, P08, P11, P12, P15, P14, P09
Arg532Phe	P16, P03, P17, P13, P08	Ile704Phe	P01, P03, P07, P13, P08, P15	Phe565Ser	P16, P18, P02, P03, P05, P06, P17, P13, P08, P11, P12, P15, P14, P09
Arg532Ser	P16, P03, P17, P13, P08	Ile704Thr	P01, P03, P07, P13, P08, P15	Phe565Val	P16, P18, P02, P03, P05, P06, P17, P13, P08, P11, P12, P15, P14, P09
Arg532Trp	P16, P03, P17, P13, P08	Leu1098_Val1101delinsProProValSer	P16, P01, P03, P07, P13, P08, P11, P12	Pro1068Ala	P16, P01, P02, P03, P05, P06, P13, P08, P11, P09, P10
Asn1071His	P16, P01, P02, P03, P05, P06, P07, P07, P13, P08, P11, P09, P10	Leu1098Pro	P16, P01, P02, P02, P03, P05, P05, P06, P06, P07, P13, P08, P11, P12, P09, P09, P10, P10	Pro1068Leu	P16, P01, P02, P03, P05, P06, P13, P08, P11, P09, P10
Asn1071Lys	P16, P01, P02, P03, P05, P06, P07, P13, P08, P11, P09, P10	Leu313Val	P07, P13, P10	Pro1088Gln	P16, P01, P02, P03, P05, P06, P07, P13, P08, P11, P09, P10
Asn1071Thr	P16, P01, P02, P03, P05, P06, P13, P08, P11, P09, P10	Leu317Val	P07, P13, P10	Pro1088Lys	P16, P01, P02, P03, P05, P06, P07, P13, P08, P11, P09, P10
Asn1074Ile	P16, P01, P02, P03, P05, P06, P07, P13, P08, P11, P09, P10	Leu504_Met505delinsHisLeu	P03, P04, P17, P08	Pro1088Thr	P16, P01, P02, P03, P05, P06, P07, P13, P08, P11, P09, P10
Asn1074Phe	P16, P01, P02, P03, P05, P06, P07, P13, P08, P11, P09, P10	Leu504Gln	P03, P04, P17, P08	Pro557_Phe558delinsArgThr	P16, P03, P13, P08, P15
Asn1074Tyr	P16, P01, P02, P03, P05, P06, P07, P13, P08, P11, P09, P10	Leu509Phe	P03, P04, P17, P08	Pro557_Phe558delinsLeuThr	P16, P03, P13, P08, P15
Asn1100Asp	P16, P01, P02, P03, P04, P06, P07, P17, P13, P08, P11, P12	Leu515Ile	P16, P03, P04, P17, P08	Pro557Leu	P16, P03, P13, P08, P15
Asn1100Ile	P16, P01, P02, P03, P06, P07, P13, P08, P11, P12	Leu515Pro	P16, P03, P04, P17, P08	Ser1069Arg	P16, P01, P02, P03, P05, P06, P13, P08, P11, P09, P10
Asn1100Lys	P16, P01, P03, P07, P13, P08, P11, P12	Leu517Val	P16, P03, P04, P17, P08	Ser1069Thr	P16, P01, P02, P03, P05, P06, P13, P08, P11, P09, P10
Asn1100Val	P02, P06	Leu522Arg	P16, P16, P03, P03, P04, P04, P17, P17, P13, P13, P08, P08	Ser1077Ala	P16, P01, P02, P03, P05, P06, P07, P13, P08, P11, P09, P10
Asn310Asp	P07, P13, P10	Leu529Met	P16, P03, P04, P17, P13, P08	Ser1077Cys	P16, P01, P02, P03, P05, P06, P07, P13, P08, P11, P09, P10
Asn500Asp	P03, P04, P17, P08	Leu529Pro	P16, P03, P04, P17, P13, P08	Ser1085Phe	P16, P01, P02, P03, P05, P06, P07, P13, P08, P11, P09, P10
Asn500Ile	P03, P04, P17, P08	Leu536_Val538delinsHisCysArg	P16, P03, P17, P13, P08	Ser1097Cys	P16, P01, P02, P03, P05, P06, P07, P13, P08, P11, P12, P09, P10
Asn500Lys	P03, P04, P17, P08	Leu536Gln	P16, P03, P17, P13, P08	Ser1097Trp	P16, P01, P02, P03, P05, P06, P07, P13, P08, P11, P12, P09, P10
Asn506His	P03, P04, P17, P08	Leu559Pro	P16, P03, P13, P08, P15	Ser497_Arg501delinsArgCysSerValIle	P03, P04, P17, P08
Asn506Ile	P03, P04, P17, P08	Leu562_Phe565delinsTerLysAspAla	P16, P18, P02, P03, P05, P06, P17, P13, P08, P11, P12, P15, P14, P09	Ser497Arg	P03, P04, P17, P08
Asn510His	P03, P04, P17, P08	Leu691His	P01, P03, P07, P13, P08, P15	Ser497Cys	P03, P04, P17, P08
Asn510Pro	P03, P04, P17, P08	Leu691Phe	P01, P03, P07, P13, P08, P15	Ser542Leu	P16, P03, P17, P13, P08
Asn510Thr	P03, P04, P17, P08	Leu691Tyr	P01, P03, P07, P13, P08, P15	Ser542Phe	P16, P03, P17, P13, P08
Asp1081Phe	P16, P01, P02, P03, P05, P06, P07, P13, P08, P11, P09, P10	Leu692Trp	P01, P03, P07, P13, P08, P15	Ser542Pro	P16, P03, P17, P13, P08
Asp1081Tyr	P16, P01, P02, P03, P05, P06, P07, P13, P08, P11, P09, P10	Lys1078_Glu1079delinsAsnLys	P16, P01, P02, P03, P05, P06, P07, P13, P08, P11, P09, P10	Ser546_Ala547delinsGlyTyr	P16, P03, P13, P08
Asp1081Val	P16, P01, P02, P03, P05, P06, P07, P13, P08, P11, P09, P10	Lys1078Asn	P16, P01, P02, P03, P05, P06, P07, P13, P08, P11, P09, P10, P12	Ser546Ala	P16, P03, P13, P08
Asp1084Val	P16, P01, P02, P03, P05, P06, P07, P13, P08, P11, P09, P10	Lys1092Asn	P16, P01, P02, P03, P05, P06, P07, P13, P08, P11, P12, P09, P10	Thr1067_Ser1069delinsThrValPro	P16, P01, P02, P03, P05, P06, P13, P08, P11, P09, P10
Asp292His	P07, P13, P10	Lys1092Glu	P16, P01, P02, P03, P05, P06, P07, P13, P08, P11, P09, P10	Thr1067Ala	P16, P01, P02, P03, P05, P06, P13, P08, P11, P09, P10
Asp499Ala	P03, P04, P17, P08	Lys1092Met	P16, P01, P02, P03, P05, P06, P07, P13, P08, P11, P09, P10	Thr1082Arg	P16, P01, P02, P03, P05, P06, P07, P13, P08, P11, P09, P10
Asp499Tyr	P03, P04, P17, P08	Lys315Thr	P07, P13, P10	Thr1082Ser	P16, P01, P02, P03, P05, P06, P07, P13, P08, P11, P09, P10
Asp526Ala	P16, P03, P04, P17, P13, P08	Lys319_Phe321delinsAsnLeuVal	P07, P13, P10	Thr1242Leu	P01, P02, P03, P13, P11, P14
Asp526Glu	P16, P03, P04, P17, P13, P08	Lys319Asn	P07, P13, P10	Thr1242Met	P16, P01, P02, P03, P07, P17, P13, P08, P11, P14, P10
Asp526Tyr	P16, P03, P04, P17, P13, P08, P01, P03, P07, P13, P08, P15	Lys319Glu	P07, P13, P10	Thr1242Pro	P01, P02, P03, P13, P11, P14
Asp696Tyr	P01, P03, P07, P13, P08, P15	Lys496_Ser497delinsLeuCys	P03, P04, P17, P08	Thr320Ile	P07, P13, P10
Asp696Val	P01, P03, P07, P13, P08, P15	Lys496Asn	P03, P04, P17, P08	Thr320Pro	P07, P13, P10
Cys555Arg	P16, P03, P13, P08, P15	Lys496Gln	P03, P04, P17, P08	Thr550Ala	P16, P03, P13, P08
Cys555Ser	P16, P03, P13, P08, P15	Lys496Met	P03, P04, P17, P08	Thr550Gly	P16, P03, P13, P08
Cys563Ser	P16, P18, P02, P03, P05, P06, P17, P13, P08, P11, P12, P15, P14, P09	Lys498Arg	P03, P04, P17, P08	Thr550Ser	P16, P03, P13, P08, P15
Cys563Trp	P16, P18, P02, P03, P05, P06, P17, P13, P08, P11, P12, P15, P14, P09	Lys498Asn	P03, P04, P17, P08	Thr552_Thr556delinsTrpLeuArgProGly	P16, P03, P13, P08, P15
Cys563Tyr	P16, P18, P02, P03, P05, P06, P17, P13, P08, P11, P12, P15, P14, P09	Lys503Arg	P03, P04, P17, P08	Thr552Ser	P16, P16, P03, P03, P13, P13, P08, P08, P15, P15
Gln344_Ile345delinsHisLeu	P04, P07, P13, P10	Lys503Asn	P03, P04, P17, P08	Thr556Ala	P16, P03, P13, P08, P15
Gln344His	P04, P07, P13, P10	Lys503Glu	P03, P04, P17, P08	Thr556Arg	P16, P03, P13, P08, P15
Gln533_Glu535delinsHisLeuGln	P16, P03, P17, P13, P08	Lys503Gly	P03, P04, P17, P08	Thr564Ala	P16, P18, P02, P03, P05, P06, P17, P13, P08, P11, P12, P15, P14, P09
Gln533His	P16, P03, P17, P13, P08	Lys513_Leu515delinsLysGlnThr	P16, P03, P04, P17, P08	Thr564Lys	P16, P18, P02, P03, P05, P06, P17, P13, P08, P11, P12, P15, P14, P09
Glu1065Asp	P16, P01, P02, P03, P05, P06, P13, P08, P11, P09, P10	Lys513Glu	P16, P03, P04, P17, P08	Trp309_Asn310delinsCysAsp	P07, P13, P10
Glu1065Gly	P16, P01, P02, P03, P05, P06, P13, P08, P11, P09, P10	Lys516Gln	P16, P03, P04, P17, P08	Trp309Arg	P07, P13, P10
Glu1079Lys	P16, P01, P02, P03, P05, P06, P07, P13, P08, P11, P09, P10	Lys525Gln	P16, P03, P04, P17, P13, P08	Trp309Cys	P07, P13, P10
Glu1089_Met1093delinsArgProGluValLeu	P16, P01, P02, P03, P05, P06, P07, P13, P08, P11, P09, P10	Lys525Met	P16, P03, P04, P17, P13, P08	Trp520Arg	P16, P03, P04, P17, P08
Glu1089Gln	P16, P01, P02, P03, P05, P06, P07, P13, P08, P11, P09, P10	Lys527Met	P16, P03, P04, P17, P13, P08	Trp553Arg	P16, P03, P13, P08, P15
Glu1089Gly	P16, P01, P02, P03, P05, P06, P07, P13, P08, P11, P09, P10	Lys537Arg	P16, P03, P17, P13, P08	Trp553Cys	P16, P03, P13, P08, P15
Glu308_Trp309delinsAspArg	P07, P13, P10	Lys537Asn	P16, P03, P17, P13, P08	Trp553Leu	P16, P03, P13, P08, P15
Glu308Asp	P07, P13, P10	Lys540_Lys541delinsCysGly	P16, P03, P17, P13, P08	Tyr1243Arg	P16, P01, P02, P03, P07, P17, P13, P08, P11, P14, P10
Glu507_Ile508delinsCysHis	P03, P04, P17, P08	Lys540Arg	P16, P03, P17, P13, P08	Tyr1243Cys	P16, P01, P02, P03, P07, P17, P13, P08, P11, P14, P10
Glu507Asp	P03, P04, P17, P08	Lys540Asn	P16, P03, P17, P13, P08	Tyr1243His	P16, P01, P02, P03, P07, P17, P13, P08, P11, P14, P10
Glu507Gly	P03, P04, P17, P08	Lys541Arg	P16, P03, P17, P13, P08	Tyr518_Ala519delinsTerThr	P16, P03, P04, P17, P08
Glu521Asp	P16, P03, P04, P17, P13, P08	Lys541Glu	P16, P03, P17, P13, P08	Tyr544His	P16, P03, P13, P08
Glu534Asp	P16, P03, P17, P13, P08	Lys697Asn	P01, P03, P07, P13, P08, P15	Tyr568Ser	P02, P03, P05, P13, P08, P09
Glu534Gln	P16, P03, P17, P13, P08	Lys697Gln	P01, P03, P07, P13, P08, P15	Val1073Leu	P16, P01, P02, P03, P05, P06, P07, P13, P08, P11, P09, P10
Glu534Val	P16, P03, P17, P13, P08	Lys697Thr	P01, P03, P07, P13, P08, P15	Val1083Gly	P16, P01, P02, P03, P05, P06, P07, P13, P08, P11, P09, P10
Glu535Gln	P16, P03, P17, P13, P08	Lys705Arg	P01, P03, P07, P13, P08, P15	Val1090Ala	P16, P01, P02, P03, P05, P06, P07, P13, P08, P11, P09, P10
Glu694Gln	P01, P03, P07, P13, P08, P15	Lys705Glu	P01, P03, P07, P13, P08, P15	Val1090Leu	P16, P01, P02, P03, P05, P06, P07, P13, P08, P11, P09, P10
Glu694Val	P01, P03, P07, P13, P08, P15	Met1086_Ile1087delinsSerPro	P16, P01, P02, P03, P05, P06, P07, P13, P08, P11, P09, P10	Val1101Ala	P16, P01, P03, P04, P07, P17, P13, P08, P11, P12
Glu699_Gly700delinsGlyHis	P01, P03, P07, P13, P08, P15	Met1086Ile	P16, P01, P02, P03, P05, P06, P07, P13, P08, P11, P09, P10	Val1101Phe	P16, P01, P03, P07, P13, P08, P11, P12
Glu699Asp	P01, P03, P07, P13, P08, P15	Met1086Leu	P16, P01, P02, P03, P05, P06, P07, P13, P08, P11, P09, P10	Val1240Gly	P01, P02, P03, P13, P11, P14
Glu699Gly	P01, P03, P07, P13, P08, P15	Met1086Thr	P16, P01, P02, P03, P05, P06, P07, P13, P08, P11, P09, P10	Val316Ala	P07, P13, P10
Gly1070_Asn1071delinsHisPro	P16, P01, P02, P03, P05, P06, P13, P08, P11, P09, P10	Met1093Ile	P16, P01, P02, P03, P05, P06, P07, P13, P08, P11, P09, P10	Val492Gly	P03, P04, P17, P08
Gly1070Arg	P16, P01, P02, P03, P05, P06, P13, P08, P11, P09, P10	Met1093Leu	P16, P01, P02, P03, P05, P06, P07, P13, P08, P11, P09, P10	Val492Met	P03, P04, P17, P08
Gly1070Glu	P16, P01, P02, P03, P05, P06, P13, P08, P11, P09, P10	Met1095Ile	P16, P01, P02, P03, P05, P06, P07, P13, P08, P11, P09, P10	Val514Glu	P16, P03, P04, P17, P08
Gly1096Arg	P16, P01, P02, P03, P05, P06, P07, P13, P08, P11, P12, P09, P10	Met1095Leu	P16, P01, P02, P03, P05, P06, P07, P13, P08, P11, P09, P10	Val514Leu	P16, P03, P04, P17, P08
Gly1096Asp	P16, P01, P02, P03, P05, P06, P07, P13, P08, P11, P12, P09, P10	Met1095Thr	P16, P01, P02, P03, P05, P06, P07, P13, P08, P11, P12, P09, P10	Val528_Leu529delinsThrThr	P16, P03, P04, P17, P13, P08
Gly1096Gln	P12	Met495Arg	P03, P03, P04, P04, P17, P17, P08, P08	Val528Ala	P16, P03, P04, P17, P13, P08
Gly1103Ala	P16, P01, P03, P04, P07, P17, P13, P08, P11, P12	Met495Leu	P03, P04, P17, P08	Val528Met	P16, P03, P04, P17, P13, P08
Gly1103Arg	P16, P01, P03, P04, P07, P17, P13, P08, P11, P12	Met505_Asn506delinsIleLeu	P03, P04, P17, P08	Val538Gly	P16, P03, P17, P13, P08
Gly1103Pro	P16, P01, P03, P04, P07, P17, P13, P08, P11, P12	Met505Ile	P03, P04, P17, P08	Val538Met	P16, P03, P17, P13, P08
Gly511Ala	P03, P04, P17, P08	Met505Leu	P03, P04, P17, P08	Val548_Gly549delinsGlyCys	P16, P03, P13, P08
Gly511Arg	P03, P04, P17, P08	Met695_Lys697delinsSerPhePro	P01, P03, P07, P13, P08, P15	Val548Gly	P16, P03, P13, P08
Gly511Pro	P03, P03, P04, P17, P08, P08	Met695Ile	P01, P03, P07, P13, P08, P15	Val554Gly	P16, P03, P13, P08, P15
Gly549Cys	P16, P03, P13, P08	Met695Leu	P01, P03, P07, P13, P08, P15	Val554Ile	P16, P03, P13, P08, P15
Gly700Arg	P01, P03, P07, P13, P08, P15	Met695Thr	P01, P03, P07, P13, P08, P15	Val698Ala	P01, P03, P07, P13, P08, P15
Gly700Glu	P01, P03, P07, P13, P08, P15	Phe1064_Thr1067delinsGlyCysThrAla	P16, P01, P02, P03, P05, P06, P13, P08, P11, P09, P10	Val702_Lys705delinsAlaLeuSerGly	P01, P03, P07, P13, P08, P15
				Val702Ala	P01, P03, P07, P13, P08, P15

## Abbreviations

ABC	ATP-binding cassette transporter
MRP	multidrug resistance-associated protein
